# Prognostic gene expression and microRNA profiling signatures and genetic alterations in primary testicular diffuse large B-cell lymphoma

**DOI:** 10.21203/rs.3.rs-5732026/v1

**Published:** 2025-01-06

**Authors:** Ken Young, Wen-Yu Shi, Zijun Xu-Monette, Youchao Jia, Alexandar Tzankov, Heounjeong Go, Ling Li, Maurilio Ponzoni, Yafei Wang, Qiongli Zhai, Anamarija Perry, Shi Wang, Xiaoxiao Wang, April Chiu, Mina Xu, Carlo Visco, Karen Dybkaer, Henry Withers, Mark Long, Alyssa Yuan, Yi Miao, Jianyong Li, Everardo Macias, Wen Shuai, Bangchen Wang, Govind Bhagat, Youli Zu, Zenggang Pan, William Choi, Santiago Montes-Moreno, Weina Chen, J. Han van Krieken, Michael Møller, Fenghuang Zhan, Ben Parsons, Shanxiang Zhang, Eric Hsi, Aliyah Sohani, Jeremy Abramson, Andrés Ferreri, Bing Xu, Yong Li

**Affiliations:** Duke University Medical Center and Duke Cancer Institute; Rui Jin hospital; Duke University School of Medicine; Duke University Medical Center; University Hospital Basel; Asan Medical Center, University of Ulsan College of Medicine; First Affiliated Hospital of Zhengzhou University; san raffaele scientific institute; Tianjin Medical University; Tianjin Cancer Center Hospital; National University Hospital, Singapore; The University of Texas MD Anderson Cancer Center; Mayo Clinic; Yale University; University of Verona; Aalborg Hospital, Aarhus University Hospital; Roswell Park Comprehensive Cancer Center; Roswell Park Comprehensive Cancer Center; Duke University Medical Center; First Affiliated Hospital of Nanjing Medical University, Jiangsu Province Hospital; First Affiliated Hospital of Nanjing Medical University, Jiangsu Province Hospital, Collaborative Innovation Center for Personalized Cancer Medicine; Duke University Medical Center; Duke University Medical Center; Duke University Medical Center; Columbia University Irving Medical Center; University of Colorado Anschutz Medical Campus; Tuen Mun Hospital; Hospital Universitario Marqués de Valdecilla/IDIVAL; UT Southwestern Medical Center; Radboud university medical center Nijmegen; Odense University Hospital; Myeloma Center, University of Arkansas for Medical Sciences; Gundersen Lutheran Health System; University of Nebraska Medical Center; Mayo Clinic; Massachusetts General Hospital; Harvard Medical School; IRCCS San Raffaele Scientific Institute; The First Affiliated Hospital of Xiamen University and Institute of Hematology, School of Medicine, Xiamen University; Baylor College of Medicine

**Keywords:** PTL, IP-LBCL, DLBCL, testis, testicular lymphoma, microRNA, profiling, gene signature, BCR, CNV, MSI, TP53 mutation, MYD88, TME, GEP, NGS, subtyping, MCD, clustering, genetic alteration, epigenetic, RNA-seq, CIBERSORT

## Abstract

Primary testicular diffuse large B-cell lymphoma (PT-DLBCL) is a rare and aggressive lymphoma with molecular heterogeneity not well characterize. In this study, we performed next-generation sequencing analysis for a large number of DNA and RNA samples from patients with PT-DLBCL. DNA sequencing analysis identified ≥ 3 chromosomes with copy number variations (CNVs) and microsatellite instability as prognostic biomarkers, rather than *MYD88* mutations and genetic subtypes. Remarkably, targeted RNA-seq analysis in 195 patients revealed that *TP53* mutations with a ≥ 40% variant allele frequency had significantly adverse prognostic impact, and that a 150-gene expression signature subdivided PT-DLBCL into two distinct clusters, termed as testicular lymphoma tumor (TLT) and microenvironment (ME) subtypes. The TLT subtype featured upregulation of genes involved in B-cell receptor signaling, cell cycle, DNA damage and repair, higher frequencies of CNVs and *MYD88* mutations, elder ages, larger tumor sizes, and significantly poorer survival. Genomic microRNA profiling analysis identified significantly differentially expressed microRNAs between 113 PT-DLBCL and 180 systemic DLBCL patients, and further subdivided the PT-DLBCL cohort by microRNA signatures. The subcohort with upregulation of 16 microRNAs associated with PT-DLBCL and testicular tissue expression had significantly better survival. This study revealed characteristic genetic, gene expression, and microRNA profiles and heterogeneity in PT-DLBCL.

## Introduction

Primary testicular (PT) diffuse large B-cell lymphoma (DLBCL) is the most common histological subtype (80–98%) of primary testicular lymphoma (PTL), an aggressive lymphoma that develops in the testes and accounts for 1–2% of non-Hodgkin lymphoma (1–3). The most recent 5th edition of World Health Organization classification of Hematolymphoid Tumors has categorized PT-DLBCL as a primary large B-cell lymphoma of immune-privileged sites (4, 5). PT-DLBCL has a high risk of central nervous system (CNS) or contralateral testicular relapse, directly conferring poor clinical outcomes historically (1, 2). Current treatments, including orchiectomy, radiotherapy, anthracycline-based chemotherapy, rituximab, and CNS-directed prophylaxis, has significantly improved the prognosis of PT-DLBCL (1, 6).

Molecularly, PT-DLBCL is predominantly of the activated B-cell-like (ABC) cell-of-origin and the MCD/C5 genetic subtypes of DLBCL (7, 8). However, PT-DLBCL demonstrates entity-specific molecular features beyond those of general DLBCL subtypes (9), as well as a unique gene pattern of methylation gain/loss (10–12) and microRNA expression compared with nodal DLBCL (13). To delineate precision medicine schemes for patients with PT-DLBCL, especially those with poor clinical outcomes, the molecular heterogeneity of PT-DLBCL must be better understood.

In this study, we performed next-generation sequencing (NGS) for DNA and RNA samples from a large cohort of patients with PT-DLBCL, clustered PT-DLBCL based on genetic, gene expression, and microRNA signatures, and analyzed for prognostic effects.

### Subjects and Method

#### Clinical samples

Diagnostic formalin-fixed paraffin-embedded tissue samples from a total of 206 PT-DLBCL patients were collected between 1993 and 2014 and achieved results by at least one type of NGS analysis in this study. The review process and clinical features of patients have been previously described (14). This study was performed in compliance with the principles of the Declaration of Helsinki and was approved as minimal to no risk or as exempt by the institutional review boards of the participating centers and Duke University.

#### DNA-sequencing and genetic subtyping

Genomic DNA samples were extracted and 100 ng of each sample was loaded to the Illumina NextSeq 550 System to sequence the exons and approximately 50 intronic nucleotides of 315 genes (15) in accordance with the manufacturer’s protocol. Sufficient reads were obtained for 192 patients with PT-DLBCL, with a median coverage of 2145x (all except 10 cases: >1000x). Sequencing data were analyzed using the DRAGEN Somatic Pipeline (Illumina) with the tumor-only analysis mode, and germline variants were removed from single-nucleotide variants (SNVs) and indels against the GRCh37 reference genome using databases and allele frequency information, as previously described (15). Chromosomal structural abnormalities and copy number variations (CNVs) against a pooled normal reference were inferred from on- and off-target sequencing reads for 188 patients using the CNVkit software (15). The LymphPlex algorithm (16) was used for clustering SNVs with a > 5% variant allele frequency (VAF) into 6 genetic subtypes: MCD-like, BN2-like, N1-like, EZB-like, ST2-like, *TP53*^mut^, and ‘not otherwise specified (NOS)’.

To evaluate tumor mutation burden (TMB) and microsatellite instability (MSI), Illumina DRAGEN-TMB and DRAGEN-MSI Pipelines were used. TMB was calculated as filtered variants (excluding driver mutations) divided by eligible regions (Mbp). MSI at a microsatellite site was called if the microsatellite repeat length distribution was significantly shifted between the tumor and normal references measured by the Jensen-Shannon distance (> 0.1, Student *t-*test p-value ≤ 0.01).

### Fluorescence in situ hybridization (FISH)

FISH analysis for *MYC*, *BCL2*, and *BCL6* alterations was performed on tissue microarrays for 200 patients with PT-DLBCL, using break-apart probes and the same methods for our systemic DLBCL samples, as described previously (17). Hybridization of *MYC*, *BCL2*, and *BCL6* failed in 34, 33, and 35 patients, respectively.

#### RNA-sequencing and data analysis

Genomic RNA was isolated and purified from each case, and 1408 cancer-associated genes were selectively enriched. Details of cDNA library construction and sequencing using the Illumina NextSeq 550 System have been described previously (18, 19). Samples that generated > 10 million reads were further analyzed. Cufflinks version 2.2.1. was used to generate an expression profile for each sample. Expression levels were measured using the Fragments Per Kilobase of transcript per Million (FPKM) method.

Gene expression profiling (GEP) data were pre-processed and normalized for 195 patients with PT-DLBCL using the Robust Multi-chip Average implemented in R package affy (release 3.16). Two-class unpaired Significance Analysis of Microarrays was performed to identify significantly differentially expressed genes (DEGs) using R package samr (version 3.0). CLUSTER 3.0 software and JAVA TREEVIEW were used to cluster and visualize gene expression. The DAVID functional annotation tool (20, 21) was used to analyze enrichment of biological processes, cellular components, molecular functions, and pathways defined by Gene Ontology (GO), UniProtKB Keywords (UP_KW), and Kyoto Encyclopedia of Genes and Genomes (KEGG).

“Cell-type Identification By Estimating Relative Subsets of RNA Transcripts” (CIBERSORT) algorithm was used to estimate the proportions of 22 tumor-infiltrating immune cells in each RNA-seq profile. Consensus clustering was perform using the unsupervised nonnegative matrix factorization (NMF) R package (22). The number of runs was set to 50, and the number of clusters was set from 3 to 10. The stability of clusters obtained via NMF was evaluated, and the previous point with the fastest decrease in the cophenetic correlation value was chosen as the optimal cluster number.

### MicroRNA profiling and comparison

The HTG EdgeSeq miRNA whole-transcriptome assay (HTG Molecular Diagnostics, Tucson, AZ) using NGS technology was used to sequence 2083 microRNAs and 13 housekeeping genes in RNA samples from patients with DLBCL, including 206 systemic and 150 primary testicular cases. Libraries were constructed and sequenced on the Illumina NextSeq platform (Illumina) as described previously (23).

Quantified data were obtained for 113 patients with PT-DLBCL and 180 patients with systemic DLBCL. Quality-controlled raw data were normalized, and variance stabilizing log2 was transformed using the DESeq2 Bioconductor package workflow (24). Differentially expressed microRNAs between two groups were identified with absolute log2 fold change > 1.0 and false discovery rate (FDR) < 0.05 (Benjamini-Hochberg corrected p-value), and displayed in heatmaps as row-scaled normalized counts.

### Statistical analysis

Overall survival (OS) was calculated from the time of diagnosis to the last follow-up or death due to any cause. Progression-free survival (PFS) was calculated from the time of diagnosis to disease progression (relapse or death) or the last follow-up. The Kaplan-Meier method and the log-rank test were used to compare OS and PFS. Quantitative data were compared using *t*-test, Mann-Whitney *U* test, or Kruskal-Wallis test. Categorical variables were compared using Fisher’s exact test. All tests were two-tailed, *P* < 0.05 was considered to be significant. Statistical analyses were performed using R package (version 4.1.2) and Graphs Prism 10, v10.2.3.

## Results

The available clinical data for the PT-DLBCL cohort with a median age of 65 years at diagnosis are summarized in Supplementary Table 1. Using the Hans algorithm (25), 139 cases (78%) were ABC, and 40 cases (22%) were germinal center B cell-like (GCB). Follow-up data were available for 183 patients. The prognostic effects of clinical parameters and ABC/GCB in this NGS cohort are shown in Supplementary Fig. 1.

### Genetic alteration analysis in PT-DLBCL reveals significant prognostic effects of chromosomal abnormalities and MSI in DNA sequences and TP53 RNA variants with high VAFs

**Figure 1:**
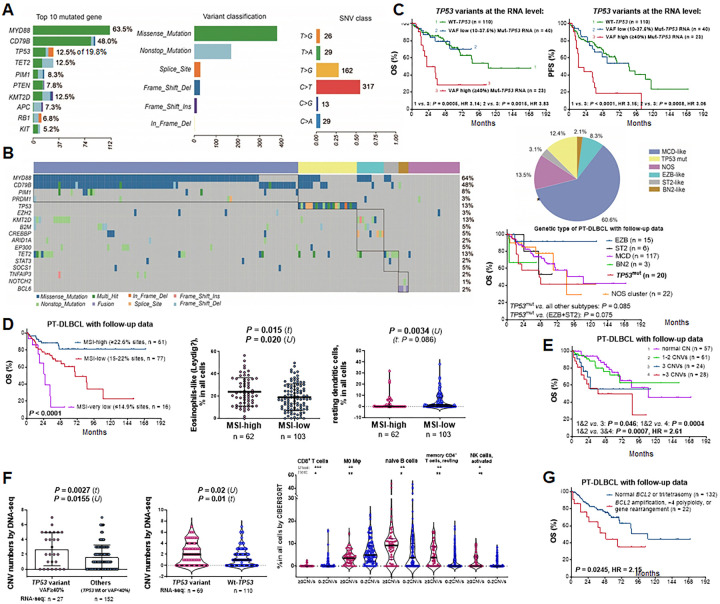
DNA sequencing results and genetic biomarkers in PT-DLBCL. **(a)** Overview of DNA mutations in 192 patients with PT-DLBCL. For *TP53* mutation, only those used for genetic subtyping were shown. (**b**) Oncoplot showing that 192 patients with PT-DLBCL were clustered into 6 genetic subtypes using the LymphPlex algorithm. MCD-like was the most frequent subtype, as shown in the pie chart. No significant prognostic effects were observed. Only the TP53^mut^ subtype showed a non-significant trend of poorer overall survival (OS). * For *TP53* mutations, only those used for LymphPlex clustering and not concurrent with subtype-predictor mutations in the MCD-like, EZB-like and ST2-like subtypes were displayed in the oncoplot. (**c**) Remarkably, presence of *TP53* mutation with a VAF≥40% by RNA-seq variant analysis was associated with significantly poorer OS and progression-free survival (PFS). (**d**) High percentages of MSI sites among evaluated sites (mostly >100) by DNA sequencing analysis were associated with significantly better survival of patients with PT-DLBCL. CIBERSORT analysis for corresponding RNA-seq data showed associations of MSI-high with higher proportion of eosinophils (possibly were Leydig cells in the testis) in the scatter plot (lines represent mean values ±SD for each group) and lower proportion of resting dendritic cells in the violin plot (lines represent medians and quantiles). (**e**) Patients with ≥3 chromosomes with copy number alterations (CNVs) had significantly poorer OS. (**f**) The scatter plot shows that *TP53* RNA variants with a VAF≥40% was associated with a high mean chromosomal CNV number in PT-DLBCL significantly by *t* test. *P* value by *U* test is also provided. The rst violin plot shows that all *TP53* variants by RNA-seq variant analysis (VAF≥10%) were associated with a higher median number of chromosomes with CNVs by DNA sequencing analysis significantly by *U* test. *P* value by *t* test is also provided. The second violin plot shows correlations of ≥3 chromosomal CNVs with lower median proportions of CD8^+^ T cells and M0 macrophages, whereas higher proportions of naïve B cells, resting memory CD4 T cells, and activated NK cells by CIBERSORT analysis. (**g**) Patients with *BCL2* gene amplification, >4 polyploid, or *BCL2* gene rearrangement by FISH analysis had significantly poorer survival than other patients with PT-DLBCL.

Targeted DNA-sequencing data were analyzed for 192 patients with PT-DLBCL. With a VAF cutoff of 5%, 114 of 315 targeted genes showed mutations. *MYD88* and *CD79B* showed high mutation frequencies in the cohort, 64% and 48%, respectively, significantly higher than those in systemic DLBCL (15)), followed by *TP53* (19.8%), *TET2* (12.5%), *KMT2D* (12.5%), *PIM1* (8.3%), *PTEN* (7.8%), *APC* (7.3%), *RB1* (6.7%), and *KIT* (5.2%) (Fig. 1A). Genetic subtyping based on mutation clusters (16) found that only the *TP53*^mut^ subtype (12.4%, excluding MCD/EZB/ST2 cases with *TP53* mutations), but not the MCD-like (61%), showed a non-significant trend of poorer OS in the cohort (Fig. 1B). Without clustering, although *TP53* variants by DNA analysis did not show a significant prognostic effect, *TP53* variants identified by RNA-seq with VAF ≥ 40% showed potent adverse impact on OS and PFS (Fig. 1C, Supplementary Fig. 2A).

TMB and MSI biomarkers were assessed for 173 patients with PT-DLBCL, and found that MSI-high, with an optimal cutoff of MSI at ≥ 22.6% of the evaluated microsatellite sites (> 100 targeted microsatellite sites were evaluated for 91.2% of cases), was associated with significantly better OS and PFS (Fig. 1D, Supplementary Fig. 2B). To examine whether MSI-high enhanced immune responses, we correlated to immune cell abundance estimated by the CIBERSORT algorithm in corresponding RNA-seq data. MSI-high patients showed significantly higher mean and median proportions of eosinophils and a lower median proportion of resting dendritic cells (Fig. 1D), trends of higher resting natural killer cells and lower M0 macrophages with borderline significance (Supplementary Fig. 2B), but not expected T cell increase. We speculated that the eosinophilic cells by CIBERSORT in this PT-DLBCL cohort were likely Leydig cells, considering the high abundance in results (26).

Large chromosomal gains or losses were present in 125 of 188 evaluated patients with PT-DLBCL, significantly more frequently than in patients with systemic DLBCL (15) (66.5% *vs*. 28.3%, *P* < 0.0001). PT-DLBCL patients with ≥ 3 chromosomal CNVs had significantly poorer OS and PFS than those without (Fig. 1F, Supplementary Fig. 2C). There were no significant differences in clinical features between two groups, except that patients with ≥ 3 chromosomal CNVs had a higher frequency of regional (but not faraway) lymph node involvement at diagnosis (44.9% *vs*. 14.3%, *P* < 0.0001), and a higher frequency of *TP53* variants identified by RNA-seq (VAF ≥ 40% variants: 24.5% *vs*. 11.2%, *P* = 0.023), and a significantly lower abundance of CD8^+^ T cells estimated by CIBERSORT (Fig. 1E). The significant association of *TP53* RNA (but not DNA) variants with elevated chromosomal CNVs in the PT-DLBCL cohort (Fig. 1E) may suggest relationships between genomic instability, *TP53* expression, and loss-of tumor suppressor function.

Using FISH analysis, *MYC*, *BCL2*, and *BCL6* gene rearrangements were found in 13 (7.8%), 4 (2.4%), and 33 (20%) patients, respectively. Three patients had dual *MYC* and *BCL2* gene (double-hit) rearrangements, including one GCB patient who was alive at the last follow-up (106 months), one GCB patient with concurrent *MYC*/*BCL2*/*BCL6* (triple hit) rearrangements who died two months after diagnosis, and a third patient without follow-up data available. Two patients had dual *MYC* and *BCL6* gene rearrangements, both alive at the last follow-up. Prognostic analysis only found that combined cases with *BCL2* amplification, high levels (> tetra) of polysomy 18, or *BCL2* rearrangement were associated with significantly poorer OS and PFS (Fig. 1G, Supplementary Fig. 2D).

### RNA-seq analysis reveals two distinct GEP clusters of PT-DLBCL with significant prognostic difference

**Figure 2:**
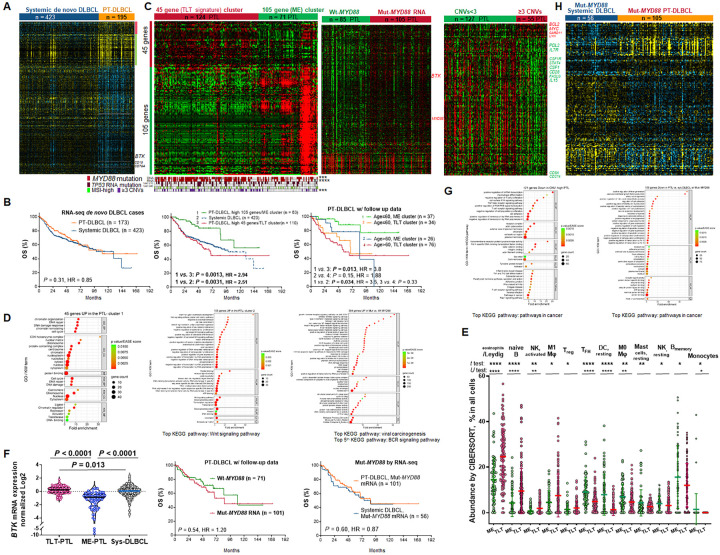
Targeted RNA-seq gene expression profiling analysis in PT-DLBCL. **(a)** Significantly differentially expressed genes (DEGs) between PT-DLBCL and systemic DLBCL patients. (**b**) The sequenced PT-DLBCL and systemic DLBCL cases did not show significant difference in overall survival (OS). The ME subtype of PT-DLBCL had significantly better OS than the TLT subtype and systemic DLBCL cases. When the age factor was integrated into the survival analysis, the favorable prognostic effect of ME subtype was significant only in patients ≤60 years old. (**c**) Left heatmap: median-centered unsupervised clustering using the 150-gene PTL signature in the PT-DLBCL cohort formed two clusters of patients (each column represents one patient) marked by horizontal red/green bars, which expressed high levels of 45 (TLT subsignature) and 105 genes (ME subsignature) of the PTL signature (each row represents expression of one gene), respectively. The vertical red/green color bars for two gene clusters on the left side of this heatmap and the similar but lighter color bars on the right side of heatmap in panel **A** indicate that the gene clusters were similar (only a few genes were different). The aligned case plot underneath the heatmap shows the status of *MYD88*mutation detected by DNA and RNA sequencing, respectively, *TP53* mutation detected by RNA sequencing with VAF≥10% and VAF≥40%, respectively, MSI-high, and ≥3 chromosomal CNVs by DNA sequencing analysis for patients in the heatmap. Significant frequency differences between two clusters are marked, ***, *P* < 0.001; ****, *P* < 0.0001. Middle heatmap: 447 DEGs between patients with and without *MYD88*mutations by RNA sequencing analysis. Right heatmap: 134 DEGs between patients with and without ≥3 chromosomes with CNVs by DNA sequencing. (**d**) Enrichment dot plots to visualize the significantly (FDR<0.05) enrichment results by DAVID functional annotation tool for 45 genes, 105 genes, and 384 genes (part of enrichment results) in the rst two heatmaps of panel **C**. The color scale shows the enrichment *P* values, horizontal axis shows fold enrichment or gene ratio, and dot sizes represents numbers of genes hits for the significant GO and KW terms. (**e**) Scatter plot showing significant differences in CIBERSORT results between the TLT and ME clusters. Each dot represents one case. Lines represent mean values ±SD. Significance: *P < 0.05; ** *P*< 0.01; *** *P* < 0.001, ****, *P* < 0.0001. (**f**) The ME subtype had significantly lower *BTK* transcription than the TLT subtype of PT-DLBCL and systemic DLBCL. The violin plot shows normalized *BTK* levels in the heatmaps in panels **A** and **C**. No significant differences in patient survival were observed between PT-DLBCL with and without *MYD88*mutation, and between PT-DLBCL and systemic DLBCL with *MYD88* mutation by RNA sequencing. (**g**) Enrichment dot plots showing part of significantly (FDR<0.05) enriched GO and KW terms and KEGG pathways for 121 genes downregulated in patients with ≥3 CNVs in the third heatmap in panel **C** and for 109 genes downregulated in PT-DLBCL *vs*. systemic DLBCL with *MYD88* mutations in panel **H**.

Expression of targeted 1408 genes by RNA-seq were compared between 195 patients with PT-DLBCL and 423 patients with systemic DLBCL (18), identifying 533 DEGs (Supplementary Table 2). When unsupervised (Supplementary Fig. 3A) instead of supervised clustering (Fig. 2A) was performed for visualization, systemic and PT-DLBCL cases were largely separated by these DEGs. Despite these molecular differences, the sequenced PT-DLBCL and systemic DLBCL patients did not show a significant difference in prognosis (Fig. 2B). However, the PT-DLBCL cohort compared with the systemic DLBCL cohort had significantly lower proportions of patients with advanced stage, B symptoms, elevated serum LDH, ECOG > 1, and International Prognostic Index > 2, whereas had a significantly higher proportion of bulky tumor (no significant differences in age and > 1 extranodal involvement), in addition to the frequency differences in gender and ABC subtype.

Notably, in Supplementary Fig. 3A and Fig. 2A, the 150 genes upregulated in PT-DLBCL (Table 1, referred to as the PTL signature) were clustered into two distinct gene groups and expressed heterogeneously in PT-DLBCL cases. To determine whether such heterogeneity had clinical relevance in PT-DLBCL, we performed median-centered unsupervised clustering within the PT-DLBCL cohort (Fig. 2C). Similar clustering was also performed using the systemic DLBCL signature (genes upregulated in systemic DLBCL; Supplementary Fig. 3B). Interestingly, two distinct clusters were formed by the PTL signature, which showed a mutually exclusive expression pattern of 45 and 105 genes, respectively (Fig. 2C), and significant differences in OS and PFS. The 105-gene cluster also had significantly better OS than the overall systemic DLBCL cohort (Fig. 2B, Supplementary Fig. 3C).

Functional annotation by the DAVID tool indicated that the 45 genes were involved in DNA repair, DNA damage response, chromatin organization and remodeling, and the cell cycle. In contrast to these intracellular processes, the 105 genes were involved in a variety of biological processes and signaling pathways (top enriched: Wnt, regulating development and stemness) more involving the tumor microenvironment (TME) and extracellular matrix (Supplementary Table 2, Fig. 2D (27)). To distinguish, the 45-gene cluster was termed as the Testicular Lymphoma Tumor (TLT) subtype, and the 105-gene cluster was termed the MicroEnvironment (ME) subtype, which is also in line with some significant differences in CIBERSORT cellularity (Fig. 2E. In addition, the ME subtype had higher CD8^+^ T cell abundance by *t* test, *P* = 0.052).

Clinically, the ME compared with the TLT subtype was associated with younger age (proportion of > 60 years old: 40% *vs*. 68.1%, *P* = 0.0003) and smaller tumor sizes (≥ 5cm: 41.8% *vs*. 69.6%, *P* = 0.0011; ≥7cm: 14.6% *vs*. 42.2%, *P* = 0.00035). No other clinical and treatment groups and ABC proportion (72.9% *vs*. 78.9%) showed significant differences between ME and TLT subtypes.

In age-matched survival analysis, in patients with age ≤ 60 years, the TLT subtype remained to show significantly poorer OS (Fig. 2B) and PFS (Supplementary Fig. 3C) than the ME subtype. Age > 60 years had a significantly adverse impact on OS in the ME subtype (Fig. 2B).

### Genetic, B-cell receptor (BCR) signaling, immune, and other factors underlie the prognostic difference between two RNA-seq subtypes of PT-DLBCL

To gain further insight into the biology of TLT and ME subtypes with significant prognostic difference, their RNA-seq GEP data were compared, revealing 264 genes upregulated in the TLT subtype and 606 genes upregulated in the ME subtype (Supplementary Fig. 3D, DAVID functional annotation is presented in Supplementary Table 3). Although *BTK* and *CD79A* were downregulated in overall PT-DLBCL compared with systemic DLBCL (therefore in the systemic DLBCL signature, Fig. 2A), the downregulation was mainly in the ME (Fig. 2F) and wild-type *MYD88* subsets (Supplementary Fig. 4A). TLT *vs*. ME subtype had upregulation of *BTK* (most significant) and BCR signaling-related genes (*LYN*, *CD79A*, *CD79B*, *SYK*, *NFKB1*, *etc.*), *PAX5*, and *MKI67* (Supplementary Fig. 3D), whereas downregulation of immune-related genes including *IL13*, *CSF1*, *IL1B*, *IL2*, *IL3*, and *CD28*. Age > 60, a clinical factor associated with the TLT subtype, was associated with 57 upregulated genes, including BCR signaling genes, *IL6* (related to infection), and *IL21R* (different from TLT *vs*. ME), and 141 downregulated genes including *EPCAM*, *AR*, *WT1*, and *CD28* (Supplementary Fig. 1C, Supplementary Table 4).

Comparing genetic alterations found that TLT had significantly higher frequencies of *MYD88* mutations and ≥ 3 chromosomal CNVs, suggesting an underlying genetic basis. Mutated *vs*. wild-type *MYD88* status was associated with upregulation of 384 genes, including 24 genes in the 45-gene TLT signature, BCR signaling genes (Supplementary Table 5, Fig. 2C, Supplementary Fig. 4B), and *MYD88* (different from TLT *vs*. ME). Different from the TLT/ME subtyping, *MYD88* status did not show significant impact on OS/PFS in patients with PT-DLBCL (Fig. 2F, Supplementary Fig. 4C).

In contrast, ≥ 3 chromosomal CNVs showed independent prognostic effects from TLT in PT-DLBCL (Supplementary Fig. 3D), and was associated with downregulation of 121 genes (Fig. 2C, Supplementary Table 6), 106 (87%) of which were also downregulated in the TLT (*vs*. ME) subtype (Supplementary Fig. 3D). DAVID functional annotation revealed several immune-related biological processes and pathways (Fig. 2G), in line with the CIBERSORT results (Fig. 1F). Likewise, tumor size ≥ 7cm was mainly associated with downregulation of 314 genes in GEP analysis (Supplementary Fig. 1C, Supplementary Table 7).

For a genetically more matched comparison between PT-DLBCL and systemic DLBCL, we restricted analysis to patients with mutated *MYD88*. *MYD88* mutated PT-DLBCL and systemic DLBCL patients showed similar prognoses (Fig. 2F, Supplementary Fig. 4C) and fewer numbers of DEGs than in the entire cohort (Fig. 2H, Supplementary Table 8, Supplementary Fig. 4D). PT-DLBCL-associated 57 genes were mainly involved in biological process in the nucleus, in contrast with 109 downregulated genes (Fig. 2G).

**Figure 3:**
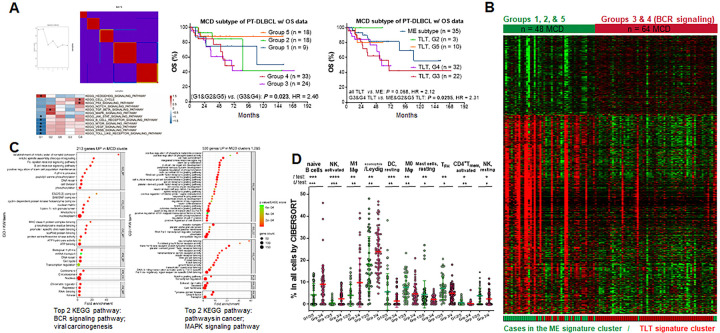
Clustering analysis in the MCD-like subtype. (**a**) Consensus matrix heatmap was based on the optimal clusters value (k=5) by cophenetic correlation coefficients for hierarchically clustered matrices. Enriched pathways in the five clusters by Gene Set Variation Analysis and multiple correction are shown in the heatmap. Significant enrichment was marked by an asterisk. Left survival curves show the OS of patients in the five GEP groups within the MCD-like genetic subtype. Right survival curves show survival analysis for effects of TLT/ME subtyping and G1-G5 grouping in patients with MCD-like genetic subtype. (**b**) Significantly differentially expressed genes between the combined G1, G2, and G5 cases and the combined G3 and G4 cases. The bar aligned below shows the TLT/ME subtypes of patients in the heatmap (each column represents one patient). (**c**) Enrichment dot plots to visualize part of significantly (FDR<0.05) enriched GO and KW terms for DEGs between combined G1, G2, and G5 cases and combined G3 and G4 cases. (**d**) The scatter plot showing significant association of G3/G4 cases with higher proportions of eosinophils-like cells, naïve B cells, activated NK cells, and M1 macrophages, and association of G1/G2/G5 cases with higher proportions of resting dendritic cells, M0 macrophages, resting mast cells, follicular helper T (TFH) cells, activated memory CD4 T cells, and resting NK cells by CIBERSORT analysis. Lines represent mean values ±SD. Significance: *P < 0.05; ** *P* < 0.01; *** *P* < 0.001, ****, *P* < 0.0001.

For a genetically more matched comparison within PT-DLBCL, we selected MCD-like PT-DLBCL cases. Unsupervised hierarchical clustering analysis was performed for their RNA-seq GEP data, dividing the MCD-like subtype into G1-G5 five clusters with different gene expression patterns (Fig. 3A). Prognostic analysis revealed that the combined G3 and G4 cases had a significantly worse OS than combined G1, G2, and G5 cases (Fig. 3A).

Subsequently, RNA-seq GEP data were compared, which revealed 213 genes upregulated in combined G3 and G4 cases and 530 genes upregulated in the combined G1, G2 and G5 cases (Fig. 3B); DAVID functional annotation is shown in Supplementary Table 9 and Fig. 3C. Cellularity differences between G3/G4 and G1/G2/G5 cases (Fig. 3D, Supplementary Fig. 5A) resembled those between the TLT and ME subtypes.

Correlating G1-G5 with TLT/ME subtyping, almost all G3/G4 cases were TLT subtype, whereas 68% of G1/G2/G5 cases were ME subtype (Fig. 3B). The G3/G4, but not G2/G5, groups of the TLT subtype had significantly poorer OS (Fig. 3A). However, ME/TLT subtyping remained to show effect on OS within the MCD-like subtype, despite the marginal significance, which may suggest that the prognostic effect of TLT *vs*. ME subtype was not restricted to the differences in genetic factors.

### MicroRNA profiling analysis identifies a set of PT-DLBCL-associated microRNAs that stratifies PT-DLBCLs with significantly better prognosis

**Figure 4:**
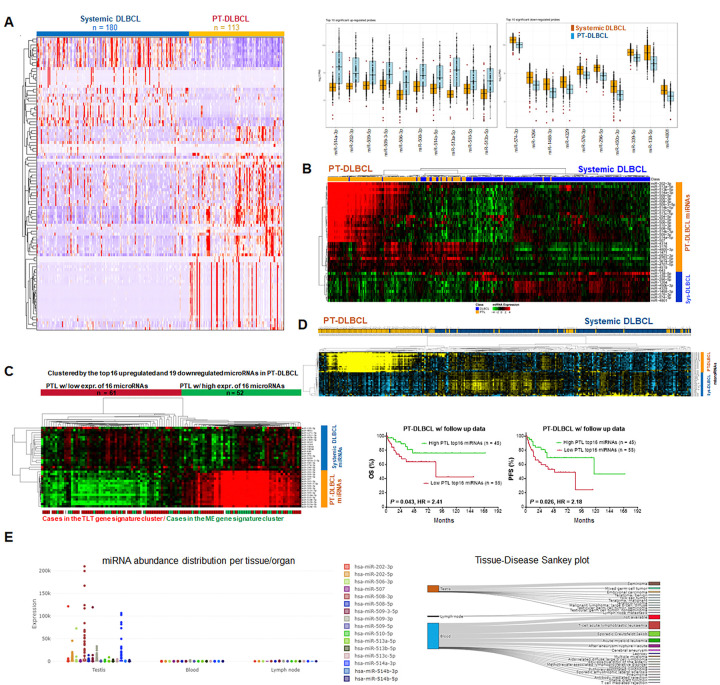
MicroRNA profiling analysis. (**a**) Heatmap showing 99 significantly differentially expressed microRNAs between sequenced systemic DLBCL and PT-DLBCL cases. Boxplots showing top 10 upregulated and top 10 downregulated microRNAs in PT-DLBCL. (**b**) Heatmap showing top 40 (including 30 up and 10 down microRNAs) significantly differentially expressed microRNAs between systemic DLBCL and PT-DLBCL. (**c**) Heatmap for expression of 35 microRNA in PT-DLBCL cases by median-centered unsupervised clustering. Patients with high expression of 16 microRNAs upregulated in PT-DLBCL *vs*. systemic DLBCL had significantly better survival than other sequenced PT-DLBCL cases. (**d**) Heatmap for expression of the 35 microRNA signature (16 upregulated and 19 downregulated microRNAs in PT-DLBCL) in PT-DLBCL and systemic DLBCL cases by unsupervised clustering. (**e**) Expression (Reads counts) of 16 microRNAs in testis, blood, and lymph nodes in the miTED database.

MicroRNAs are short single-stranded non-coding RNAs that can epigenetically regulate the expression of multiple genes (28). We performed microRNA profiling for PT-DLBCL and systemic DLBCL cohorts and compared between two cohorts. Figure 4A shows a heatmap of 66 upregulated and 33 downregulated microRNAs in PT-DLBCL and boxplots of top up/down microRNAs. As shown in Supplementary Fig. 5B, the differentially expressed microRNAs formed clusters expressed in different patient subsets by unsupervised clustering. To identify microRNA clusters with prognostic role in PT-DLBCL, we performed unsupervised clustering in the PT-DLBCL cohort using different microRNAs sets, including the 99 significant microRNAs, top 60 microRNAs, top 40 microRNAs (Fig. 4B), the combined top 10 upregulated and top 10 downregulated microRNAs, and the combined top 20 upregulated and top 20 downregulated microRNAs (Supplementary Fig. 5C). significant prognostic effects were shown by grouping patients based on 16 microRNAs (miR-514a-3p, miR-202–3p, miR-509–5p, miR-509–3-5p, miR-506–3p, miR-508–3p, miR-514b-5p, miR-513a-5p, miR-510–5p, miR-513b-5p, miR-513c-5p, miR-508–5p, miR-509–3p, miR-202–5p, miR-507, and miR-514b-3p) from those upregulated in PT-DLBCL compared with systemic DLBCL. Subsequently, four upregulated and one downregulated microRNAs were removed from the 40-microRNA signature in Supplementary Fig. 5C, and unsupervised clustering were performed in the PT-DLBCL cohort (Fig. 4C) and in all assessed DLBCL cases (Fig. 4D).

To understand the biology underlying the prognostic effect, we compared RNA-seq GEP data of patient groups in Fig. 4C. Although no DEGs showed FDR ≤ 5, there were 10 genes with q < 0.001 and 45 genes with q = 0.056 downregulated in the 16 microRNAs-high group (Supplementary Fig. 5D), suggesting epigenetic suppression. However, *DICER1* gene, which encodes the Dicer protein indispensable for microRNA biogenesis, was among these downregulated genes (q < 0.001, FDR 0.092), which appeared to be more relevant for the downregulation of systemic DLBCL-associated microRNAs in this patient group. Comparing clinical features of the two groups only found that the microRNA-high group had significant lower proportion of patients with tumor size ≥ 7cm (18% *vs*. 51%, *P* = 0.0017) and patients over 75 years old (8.33% *vs*. 32.14%, *P* = 0.043).

We used the web-based microRNA Tissue Expression Database (miTED, https://dianalab.e-ce.uth.gr/mited/#/expressions) (28) collected from two repositories (SRA and TCGA) to gain insights into the potential cell resources of the top 20 upregulated and 20 downregulated microRNAs in PT-DLBCL compared with systemic DLBCL. Distinctly, 14 of the 16 upregulated microRNAs (but not the 4 microRNAs excluded from our nal microRNA signature) showed testicular tissue specificity, expressed in testicular disease samples but not in lymph nodes and blood samples (medians were 0) in the database. In contrast, about half of the downregulated microRNAs had higher expression in lymph nodes and/or blood samples than in testicular tissues (Fig. 4E, Supplementary Fig. 6).

## Discussion

In this study, we simultaneously analyzed DNA and RNA samples of a large PT-DLBCL cohort using NGS, delineated the mutational landscapes and expression profiles of genomic microRNAs and 1408 genes in PT-DLBCL compared with systemic DLBCL, and subdivided PT-DLBCLs into NGS clusters with significant prognostic differences.

DNA-sequencing identified CNV-high and MSI-high biomarkers with significantly unfavorable and favorable, respectively, prognostic effects in PT-DLBCL, rather than *MYD88*/*CD79B* mutations and the MCD genetic subtype (2, 16, 29). By FISH analysis, *MYC* and *BCL2* gene rearrangements were infrequent, as PT-DLBCLs were mostly of non-GCB; however, poorer prognosis was observed in subset of cases with *BCL2* abnormalities. While *TP53* mutations in 23% of patients by DNA-sequencing did not show a significant prognostic effect, simultaneous RNA-seq detected *TP53* variants in 38.8% of patients and associated with elevated chromosomal CNVs, and those with ≥ 40% VAFs demonstrated remarkably adverse prognostic effects in patients with PT-DLBCL. In contrast, DNA-sequencing detected more variants than RNA-seq for *MYD88* mutations in this PT-DLBCL cohort (Fig. 2C) and for *MYD88* and *TP53* variants in our systemic DLBCL cohort (15, 18), which had lower frequencies of bulky tumors and CNVs than this PT-DLBCL cohort. Further studies could investigate the p53 protein expression in PT-DLBCL and novel therapies for the *TP53*^mut^ subtype, such as adding decitabine, a DNA methylation inhibitor, to the standard of care, which improved the clinical outcomes of *TP53*^mut^ patients with systemic DLBCL in a phase II clinical trial (30).

GEP analysis for targeted RNA-seq data identified two distinct subtypes of PT-DLBCL, TLT and ME, with the ME subtype having significantly better OS than TLT-PTL and systemic DLBCL. The ME-PTL subsignature showed higher specificity for favorable prognostic impact (Fig. 2) than the 200-gene subcluster of the systemic DLBCL signature by unsupervised clustering (Supplementary Fig. 3B). The TLT subtype, clustered by 45 genes involved in the cell cycle and DNA damage and repair, had higher expression of B-cell markers, *BTK*, BCR signaling genes, and *MKI67*, suggesting proliferative tumor cells as the cell resource. The TLT subtype was associated with elderly ages, bulky tumors, *MYD88* mutations, and elevated CNVs; however, these clinical and genetic factors, which were not associated with each other, may only dissect the TLT subtype but not account for all its biological features and prognostic effects. In contrast, the ME subtype and wild-type *MYD88* PT-DLBCL had significantly lower *BTK* expression than systemic DLBCL, informative for BTK inhibitor therapies (30). However, as our RNA-seq was bulk profiling, a higher proportion of TME cells in samples could also exhibit lower *BTK* levels. Notable upregulated signaling pathways shown in the ME subtype included Wnt signaling, Hedgehog signaling (31) in the G1 subcluster, TGF- β, NOTCH, and MAPK signaling in the G2 subcluster, and JAK-STAT signaling in the G5 subcluster of MCD-like cases.

Genomic microRNA profiling analysis also suggested a favorable role of the TME in PT-DLBCL. Most of the 16 microRNAs upregulated in the prognosis-favorable patient cluster showed testicular tissue specificity but they were not microRNAs specific for germ cell tumors (32), suggesting that testicular-specific TME harbored favorable prognostic factors. Somewhat contradictory, the prognosis-favorable ME subtype of PT-DLBCL showed less eosinophils by CIBERSORT analysis, which we speculated as Leydig cells. Leydig cell abundance can affect the blood-testis barrier and immune privilege (33, 34). We acknowledge the limitations of this study in bulk profiling and retrospective nature with incomplete clinical and follow-up data and non-uniformed treatment. To pinpoint the favorable factors in the TME would require single-cell expression analysis. Currently available single-cell data in PT-DLBCL included single-cell RNA-seq for only three patients (35) and multiplex immunohistochemistry for several immune cell markers in 60–80 patients (9, 36, 37).

In summary, this NGS study identified distinctive genetic, transcriptional, and microRNA characteristics of PT-DLBCL compared with systemic DLBCL, and further revealed clinically relevant heterogeneity in PT-DLBCL. Results suggest that highly proliferative testicular lymphoma cells with enhanced cell cycle, elevated genomic stress, activated BCR signaling, and likely immune escape mechanisms are drivers of aggressive PT-DLBCL and confer poor clinical outcome; in contrast, the unique TME in PT-DLBCL harbors antitumor factors conferring better prognosis, especially in younger patients. Our novel NGS-based findings are informative for risk stratification and future mechanistic and therapeutic studies in PT-DLBCL.

## Figures and Tables

**Tale 1.** Genes in the primary testicular lymphoma GEP signature, grouped into the 45-gene LTL subsignature and the 105-gene ME subsignature

	45 genes	105 genes

Gene name	*CDK8, CDK4, DEK BCL2, GAB1, AKAP9, FBXW7, CCND2, CREB3L2, TERT, CHEK1, BRCA1, BRCA2, CENPF, KIAA1524, SUV39H2, FANCM, BCL11A TCL1A, BAP1, CAD, RECQL4, WDR90, RASGRF1, PIM1, IRF4, KTN1, SMC3, BUB1B, IKZF1, PDCD11, TBL1XR1, ABU, CARD11, GRHPR, FOXP1, MLLT4, KMT2D, BAZ2A, MDC1, MIB1, CIRH1A, RASAL1, TP63, ANKRD28*	*EPCAM, EPHB1, FRK, CEBPD, CKB, RUNX1T1, BCL2L2, TGFBR3, AMH, HLF, PAX8, PLA2G5, IGFBP2, FHL2, MYH11, WT1, SMARCA1, DAB2IP, FSTL3, FZD3, COL9A3, FANCF, WNT3, WNT10B, ZIC2, WNT6, NGFR, BCL9, DUSP9, DACH1, ITPKA, HOXD9, PPP3R2, DMRT1, NCAM1, AR, CNTN1, ERBB4, PEG3, LAMA1, DLEC1, NAV3, ARHGAP20, MN1, NTRK1, GLI1, WNT7B, EPO, EGR4, FOXL2, DMRTA2, GDF6, FEV, DUX2, GFAP, SOX2, ID4, LEFTY2, PRKCG, SOX10, HNF1A, HOXA11, NKX2–5, TLX1, RYR3, PRKACG, HOXC13, PAK7, PRMT8, LRRC7, NUTM1, LINGO2, TRHDE, EPHA10, C10orf55, WIF1, WNT4, SRRM3, RNF43, TLX3, CXXC4, SH2D5, KIAA1549, DIRAS3, ACSBG1, MAPK8IP2, IRS2, OLIG1, DLL3, USP6, PTCRA, RERG, PHOX2B, PAK6, FAM19A5, DUX4L4, ZNF444, NEURL1, ATP8A2, FZD6, PRICKLE1, CCT6B, DDX20, DCLK2, MALAT1*

## Data Availability

The analyzed data supporting the conclusions of this study are included in the figures and additional files. The datasets used in the current study are available from the corresponding author upon reasonable request.

## References

[1] Pollari M, Leivonen SK, Leppä S. Testicular Diffuse Large B-Cell Lymphoma-Clinical, Molecular, and Immunological Features. Cancers (Basel). 2021;13(16). 10.3390/cancers13164049 PMC8392512 34439203

[2] Twa DDW, Mottok A, Savage KJ, Steidl C. The pathobiology of primary testicular diffuse large B-cell lymphoma: Implications for novel therapies. Blood Rev. 2018;32(3):249–55. 29289361 10.1016/j.blre.2017.12.001

[3] Chen B, Cao DH, Lai L, Guo JB, Chen ZY, Huang Y, Adult primary testicular lymphoma: clinical features and survival in a series of patients treated at a high-volume institution in China. BMC Cancer. 2020;20(1):220. 32171265 10.1186/s12885-020-6711-0 PMC7071578

[4] Alaggio R, Amador C, Anagnostopoulos I, Attygalle AD, Araujo IBO, Berti E, The 5th edition of the World Health Organization Classification of Haematolymphoid Tumours: Lymphoid Neoplasms. Leukemia. 2022;36(7):1720–48. 35732829 10.1038/s41375-022-01620-2 PMC9214472

[5] RoschewskiM, PhelanJD, JaffeES. Primary Large B-cell Lymphomas of Immune-Privileged Sites. Blood. 2024.10.1182/blood.202302091138635786

[6] Cheah CY, Wirth A, Seymour JF. Primary testicular lymphoma. Blood. 2014;123(4):486–93. 24282217 10.1182/blood-2013-10-530659

[7] Chapuy B, Stewart C, Dunford AJ, Kim J, Kamburov A, Redd RA, Molecular subtypes of diffuse large B cell lymphoma are associated with distinct pathogenic mechanisms and outcomes. Nature medicine. 2018;24(5):679–90. 10.1038/s41591-018-0016-8 PMC6613387 29713087

[8] Wright GW, Huang DW, Phelan JD, Coulibaly ZA, Roulland S, Young RM, A Probabilistic Classification Tool for Genetic Subtypes of Diffuse Large B Cell Lymphoma with Therapeutic Implications. Cancer Cell. 2020;37(4):551–68 e14. 32289277 10.1016/j.ccell.2020.03.015 PMC8459709

[9] Autio M, Brück O, Pollari M, Karjalainen-Lindsberg ML, Beiske K, Jørgensen JM, Characterization of tumor microenvironment and cell interaction patterns in testicular and diffuse large B-cell lymphomas. Haematologica. 2024. 10.3324/haematol.2024.286267 PMC12130789 39665214

[10] Kawakami T, Okamoto K, Kataoka A, Koizumi S, Iwaki H, Sugihara H, Multipoint methylation analysis indicates a distinctive epigenetic phenotype among testicular germ cell tumors and testicular malignant lymphomas. Genes Chromosomes Cancer. 2003;38(1):97–101. 12874790 10.1002/gcc.10234

[11] Shen Y, Ou J, He B, Yang J, Liu H, Wang L, 5-Hydroxymethylation alterations in cell-free DNA reflect molecular distinctions of diffuse large B cell lymphoma at different primary sites. Clin Epigenetics. 2022;14(1):126. 36221115 10.1186/s13148-022-01344-1 PMC9555108

[12] Shen Y, Wang L, Ou J, Wang B, Cen X. Loss of 5-hydroxymethylcytosine as a Poor Prognostic Factor for Primary Testicular Diffuse Large B-cell Lymphoma. Int J Med Sci. 2022;19(2):225–32. 35165508 10.7150/ijms.65517 PMC8795795

[13] Mansoor A, Akhter A, Shabani-Rad MT, Deschenes J, Yilmaz A, Trpkov K, Primary testicular lymphoma demonstrates overexpression of the Wilms tumor 1 gene and different mRNA and miRNA expression profiles compared to nodal diffuse large B-cell lymphoma. Hematol Oncol. 2023;41(5):828–37. 37291944 10.1002/hon.3190

[14] Deng L, Xu-Monette ZY, Loghavi S, Manyam GC, Xia Y, Visco C, Primary testicular diffuse large B-cell lymphoma displays distinct clinical and biological features for treatment failure in rituximab era: a report from the International PTL Consortium. Leukemia. 2016;30(2):361–72. 26308769 10.1038/leu.2015.237

[15] Xu-Monette ZY, Wei L, Fang X, Au Q, Nunns H, Nagy M, Genetic Subtyping and Phenotypic Characterization of the Immune Microenvironment and MYC/BCL2 Double Expression Reveal Heterogeneity in Diffuse Large B-cell Lymphoma. Clinical cancer research: an official journal of the American Association for Cancer Research. 2022;28(5):972–83. 34980601 10.1158/1078-0432.CCR-21-2949 PMC9137388

[16] Shen R, Fu D, Dong L, Zhang MC, Shi Q, Shi ZY, Simplified algorithm for genetic subtyping in diffuse large B-cell lymphoma. Signal Transduct Target Ther. 2023;8(1):145. 37032379 10.1038/s41392-023-01358-y PMC10083170

[17] Tzankov A, Xu-Monette ZY, Gerhard M, Visco C, Dirnhofer S, Gisin N, Rearrangements of MYC gene facilitate risk stratification in diffuse large B-cell lymphoma patients treated with rituximab-CHOP. Modern pathology: an official journal of the United States and Canadian Academy of Pathology, Inc. 2014;27(7):958–71. 24336156 10.1038/modpathol.2013.214

[18] Xu-Monette ZY, Zhang H, Zhu F, Tzankov A, Bhagat G, Visco C, A refined cell-of-origin classifier with targeted NGS and artificial intelligence shows robust predictive value in DLBCL. Blood Adv. 2020;4(14):3391–404. 32722783 10.1182/bloodadvances.2020001949 PMC7391158

[19] Albitar M, Zhang H, Goy A, Xu-Monette ZY, Bhagat G, Visco C, Determining clinical course of diffuse large B-cell lymphoma using targeted transcriptome and machine learning algorithms. Blood Cancer J. 2022;12(2):25. 35105854 10.1038/s41408-022-00617-5 PMC8807629

[20] Sherman BT, Hao M, Qiu J, Jiao X, Baseler MW, Lane HC, DAVID: a web server for functional enrichment analysis and functional annotation of gene lists (2021 update). Nucleic acids research. 2022;50(W1):W216–W21. 35325185 10.1093/nar/gkac194 PMC9252805

[21] Huang da W, Sherman BT, Lempicki RA. Systematic and integrative analysis of large gene lists using DAVID bioinformatics resources. Nat Protoc. 2009;4(1):44–57. 19131956 10.1038/nprot.2008.211

[22] Brunet JP, Tamayo P, Golub TR, Mesirov JP. Metagenes and molecular pattern discovery using matrix factorization. Proc Natl Acad Sci U S A. 2004;101(12):4164–9. 15016911 10.1073/pnas.0308531101 PMC384712

[23] Zhou H, Xu-Monette ZY, Xiao L, Strati P, Hagemeister FB, He Y, Prognostic factors, therapeutic approaches, and distinct immunobiologic features in patients with primary mediastinal large B-cell lymphoma on long-term follow-up. Blood Cancer J. 2020;10(5):49. 32366834 10.1038/s41408-020-0312-7 PMC7198569

[24] Love MI, Huber W, Anders S. Moderated estimation of fold change and dispersion for RNA-seq data with DESeq2. Genome Biol. 2014;15(12):550. 25516281 10.1186/s13059-014-0550-8 PMC4302049

[25] Hans CP, Weisenburger DD, Greiner TC, Gascoyne RD, Delabie J, Ott G, Confirmation of the molecular classification of diffuse large B-cell lymphoma by immunohistochemistry using a tissue microarray. Blood. 2004;103(1):275–82. 14504078 10.1182/blood-2003-05-1545

[26] Aladamat N, Tadi P. Histology, Leydig Cells. 2024.

[27] Tang D, Chen M, Huang X, Zhang G, Zeng L, Zhang G, SRplot: A free online platform for data visualization and graphing. PloS one. 2023;18(11):e0294236. 37943830 10.1371/journal.pone.0294236 PMC10635526

[28] Kavakiotis I, Alexiou A, Tastsoglou S, Vlachos IS, Hatzigeorgiou AG. DIANA-miTED: a microRNA tissue expression database. Nucleic acids research. 2022;50(D1):D1055–D61. 34469540 10.1093/nar/gkab733 PMC8728140

[29] Booman M, Douwes J, Glas AM, de Jong D, Schuuring E, Kluin PM. Primary testicular diffuse large B-cell lymphomas have activated B-cell-like subtype characteristics. J Pathol. 2006;210(2):163–71. 16823896 10.1002/path.2033

[30] Zhang MC, Tian S, Fu D, Wang L, Cheng S, Yi HM, Genetic subtype-guided immunochemotherapy in diffuse large B cell lymphoma: The randomized GUIDANCE-01 trial. Cancer Cell. 2023;41(10):1705–16.e5. 37774697 10.1016/j.ccell.2023.09.004

[31] Ramirez E, Singh RR, Kunkalla K, Liu Y, Qu C, Cain C, Defining causative factors contributing in the activation of hedgehog signaling in diffuse large B-cell lymphoma. Leuk Res. 2012;36(10):1267–73. 22809693 10.1016/j.leukres.2012.06.014 PMC3422406

[32] Belge G, Grobelny F, Radtke A, Bodes J, Matthies C, Wülfing C, Serum levels of microRNA-371a-3p are not elevated in testicular tumours of non-germ cell origin. J Cancer Res Clin Oncol. 2021;147(2):435–43. 33200255 10.1007/s00432-020-03429-x PMC7817581

[33] Ollila TA, Olszewski AJ. Extranodal Diffuse Large B Cell Lymphoma: Molecular Features, Prognosis, and Risk of Central Nervous System Recurrence. Curr Treat Options Oncol. 2018;19(8):38. 29931605 10.1007/s11864-018-0555-8 PMC6294323

[34] Justin Margret J, Jain SK. L-Cysteine Upregulates Testosterone Biosynthesis and Blood-Testis Barrier Genes in Cultured Human Leydig Cells and THP-1 Monocytes and Increases Testosterone Secretion in Human Leydig Cells. Biomolecules. 2024;14(9). 10.3390/biom14091171 PMC11430594 39334937

[35] Bian Z, Gu B, Shi G, Guo J, Li D, Zeng H, The single-cell landscape reveals unique tumor subsets and microenvironments associated with poor clinical outcomes in primary testicular diffuse large B-cell lymphoma. Genes Dis. 2024;11(1):80–3. 37588237 10.1016/j.gendis.2023.02.036 PMC10425787

[36] Leivonen SK, Pollari M, Brück O, Pellinen T, Autio M, Karjalainen-Lindsberg ML, T-cell inflamed tumor microenvironment predicts favorable prognosis in primary testicular lymphoma. Haematologica. 2019;104(2):338–46. 30237271 10.3324/haematol.2018.200105 PMC6355505

[37] Pollari M, Brück O, Pellinen T, Vähämurto P, Karjalainen-Lindsberg ML, Mannisto S, PD-L1(+) tumor-associated macrophages and PD-1(+) tumor-infiltrating lymphocytes predict survival in primary testicular lymphoma. Haematologica. 2018;103(11):1908–14. 30026337 10.3324/haematol.2018.197194 PMC6278972

